# Organosolv pretreatment assisted by carbocation scavenger to mitigate surface barrier effect of lignin for improving biomass saccharification and utilization

**DOI:** 10.1186/s13068-021-01988-w

**Published:** 2021-06-12

**Authors:** Qiulu Chu, Wenyao Tong, Jianqiang Chen, Shufang Wu, Yongcan Jin, Jinguang Hu, Kai Song

**Affiliations:** 1 Jiangsu Co-Innovation Center of Efficient Processing and Utilization of Forest Resources, College of Light Industry and Food Engineering, Nanjing Forestry University, No.159 Longpan Road, Nanjing, 210037 China; 2 College of Biology and the Environment, Nanjing Forestry University, No.159 Longpan Road, Nanjing, 210037 China; 3 International Innovation Center for Forest Chemicals and Materials, Nanjing Forestry University, Nanjing, 210037 China; 4 Department of Chemical and Petroleum Engineering, University of Calgary, 2500 University Dr. NW, Calgary, AB T2N 1Z4 Canada

**Keywords:** Ethanol organosolv pretreatment, Lignocellulosic biomass, Carbocation scavenger, Enzymatic hydrolysis, Sugar production, Lignin adsorbents

## Abstract

**Background:**

Ethanol organosolv (EOS) pretreatment is one of the most efficient methods for boosting biomass saccharification as it can achieve an efficient fractionation of three major constituents in lignocellulose. However, lignin repolymerization often occurs in acid EOS pretreatment, which impairs subsequent enzymatic hydrolysis. This study investigated acid EOS pretreatment assisted by carbocation scavenger (2-naphthol, 2-naphthol-7-sulfonate, mannitol and syringic acid) to improve biomass fractionation, coproduction of fermentable sugars and lignin adsorbents. In addition, surface barrier effect of lignin on cellulose hydrolysis was isolated from unproductive binding effect of lignin, and the analyses of surface chemistry, surface morphology and surface area were carried out to reveal the lignin inhibition mitigating effect of various additives.

**Results:**

Four different additives all helped mitigate lignin inhibition on cellulose hydrolysis in particular diminishing surface barrier effect, among which 2-naphthol-7-sulfonate showed the best performance in improving pretreatment efficacy, while mannitol and syringic acid could serve as novel green additives. Through the addition of 2-naphthol-7-sulfonate, selective lignin removal was increased up to 76%, while cellulose hydrolysis yield was improved by 85%. As a result, 35.78 kg cellulose and 16.63 kg hemicellulose from 100 kg poplar could be released and recovered as fermentable sugars, corresponding to a sugar yield of 78%. Moreover, 22.56 kg ethanol organosolv lignin and 17.53 kg enzymatic hydrolysis residue could be recovered as lignin adsorbents for textile dye removal, with the adsorption capacities of 45.87 and 103.09 mg g^−1^, respectively.

**Conclusions:**

Results in this work indicated proper additives could give rise to the form of less repolymerized surface lignin, which would decrease the unproductive binding of cellulase enzymes to surface lignin. Besides, the supplementation of additives (NS, MT and SA) resulted in a simultaneously increased surface area and decreased lignin coverage. All these factors contributed to the diminished surface barrier effect of lignin, thereby improving the ease of enzymatic hydrolysis of cellulose. The biorefinery process based on acidic EOS pretreatment assisted by carbocation scavenger was proved to enable the coproduction of fermentable sugars and lignin adsorbents, allowing the holistic utilization of lignocellulosic biomass for a sustainable biorefinery.

**Graphic abstract:**

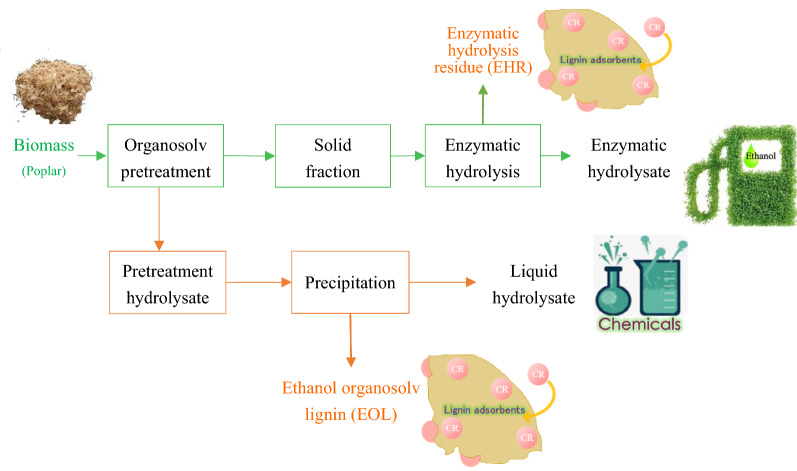

**Supplementary Information:**

The online version contains supplementary material available at 10.1186/s13068-021-01988-w.

## Highlights

- An acid organosolv pretreatment assisted by carbocation scavenger was developed.
- Additives helped improve biomass fractionation, saccharification and utilization.
- The surface barrier effect of lignin on cellulose hydrolysis was notably diminished.
- Up to 78% of carbohydrate was released and could be recovered as fermentable sugars.
- Lignin was recovered as adsorbents for Congo red removal from wastewater.

## Background

Biorefinery, an alternative technique to traditional petroleum refinery, aims at utilization of renewable lignocellulosic biomass to produce bio-based fuels, chemicals and materials [1, 2]. In lignocellulosic biomass, cellulose and hemicellulose are considered as the economical constituents which are amenable to produce fermentable sugars and further be converted to biofuels and chemicals [3, 4]. However, the presence of lignin in lignocellulosic biomass and the resulting complex lignocellulosic matrix make it highly resistant towards saccharification by microorganisms or enzymes to produce fermentable sugars [5]. Thereby, a pretreatment stage is always essential to open up lignocellulosic matrix by fractionating lignin and/or partial hemicellulose components, making cellulose more available to enzymatic attack [6, 7].

Among various pretreatment, acidic pretreatment (e.g., dilute acid pretreatment, liquid hot water pretreatment) mostly promotes hemicellulose solubilization, resulting in improved ease of enzymatic hydrolysis of cellulose. However, lignin is not fully removed during acidic pretreatment, which would redeposit back onto fiber surface and restrict the cellulose accessibility to cellulase enzymes [8]. Besides, lignin also reduces the availability and activity of enzymes during hydrolysis through unproductive binding [9]. It is suggested that ethanol organosolv (EOS) pretreatment, in particular with acid catalyst, can effectively remove lignin and hemicellulose together through the cleaving of the lignin–carbohydrate complex, yielding cellulose-enriched solids with improved ease of enzymatic hydrolysis [1, 3]. After acid organosolv pretreatment and subsequent enzymatic hydrolysis, lignocellulosic biomass can be effectively fractionated into cellulosic sugars, hemicellulosic sugars and organosolv lignin [10]. As reported, the cellulosic and hemicellulosic sugars can be efficiently converted to bioethanol and value-added chemicals [3, 4]. In the case of derived lignin, it offers promising opportunities to use lignin as antioxidants [11], adsorbents [12] and other bio-based materials [13, 14].

However, during acid EOS pretreatment, repolymerization reactions of lignin often occur, through carbocation-induced condensation [7, 15] and radical-induced coupling of lignin fragments [1]. Repolymerization reactions cause a form of more condensed and less hydrophilic lignin structure, which aggravates the hydrophobic interactions between lignin and enzymes in subsequent enzymatic hydrolysis [16]. Besides, phenolic hydroxyl groups (PhOH) are generated, in which the condensed PhOH exhibit strong association with lignin inhibition on cellulose hydrolysis, due to the hydrogen bonding interactions of lignin to enzymes [17]. Therefore, lignin repolymerization causes extra lignin inhibition on enzymatic hydrolysis of cellulose.

It has been proposed that proper additives in pretreatment that suppress lignin repolymerization have the potential to improve biomass saccharification [18, 19]. Some additives such as syringic acid and 2-naphthol have been reported to effectively scavenge the carbocation and/or radical intermediates, thereby enhancing the enzymatic hydrolysis yield of dilute acid or autohydrolysis (e.g., liquid hot water or steam explosion) pretreated softwood by up to 64% [20–22]. Besides, mannitol, as an aliphatic alcohol, is believed to help alleviate lignin repolymerization by mainly forming Cα-etherified lignin with plenty hydroxyl tails, like other reported aliphatic alcohols (methanol, ethanol and 1,4-butanediol) [16, 18]. Moreover, mannitol can specifically quench •OH radicals that are present where hydrothermal reactions take place using water and solvent [23], which has the potential to reduce the radical coupling of lignin fragments. By suppressing lignin repolymerization, lignin inhibition including unproductive binding and surface barrier effect is expected to be mitigated, for improved ease of enzymatic conversion of carbohydrates. To the best of the authors’ knowledge, this is the first study to utilize these additives in acid EOS pretreatment of hardwood for a comprehensive evaluation on the strategy of suppressing lignin repolymerization for boosting enzymatic saccharification of lignocellulose.

In this work, poplar wood sawdust, as a typical hardwood, was initially subjected to an acid EOS pretreatment, aiming at fractionation of lignin to improve cellulose accessibility and enzymatic digestibility. Subsequently, the effect of additives (2-naphthol, 2-naphthol-7-sulfonate, mannitol and syringic acid) in acid EOS pretreatment was compared on facilitating biomass fractionation and enhancing the ease of cellulose hydrolysis. Moreover, it is of significance to find a way to not only fractionate lignicellulosic components for efficient sugar production, but also produce value-added materials for economic consideration from the view of potential industrialization. Thus, in this work the organosolv lignin, as well as the lignin-containing residues after enzymatic hydrolysis, were recovered and utilized as adsorbents of textile dyes removal for wastewater treatment. Finally, the scheme of mass balance analysis of biorefinery based on the acid EOS pretreatment assisted by carbocation scavenger were carried out, in order to evaluate the process efficiency to coproduce fermentable sugars and lignin materials from highly recalcitrant woody biomass.

## Results and discussion

### Effect of acid concentration on pretreatment efficacy

**Fig. 1:**
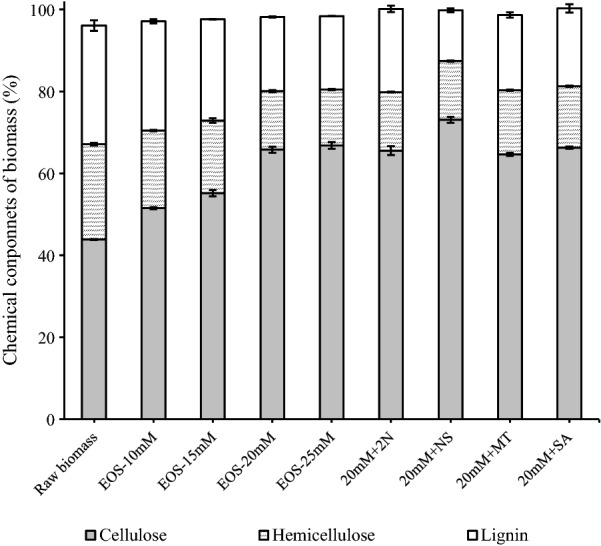
Effect of acid concentration (10–25 mM) and additives on chemical components of the acid EOS pretreated substrates (*EOS* ethanol organosolv pretreatment, *2N* 2-naphthol, *NS* 2-naphthol-7-sulfonate, *MT* mannitol, *SA* syringic acid)

**Fig. 2:**
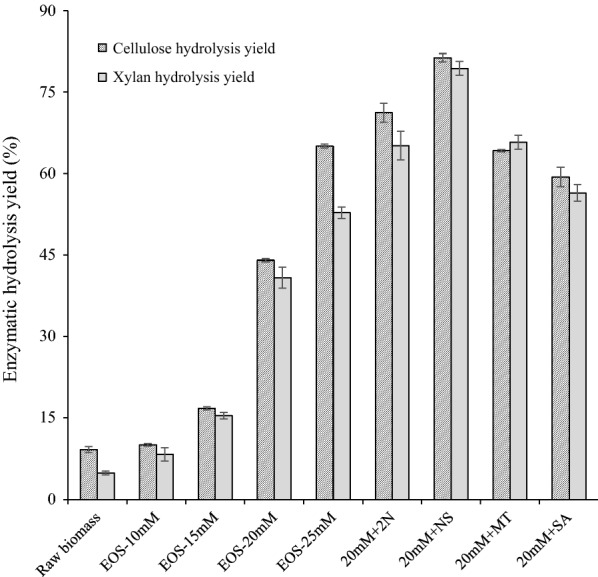
Effect of acid concentration (10–25 mM) and various additives on enzymatic hydrolysis yield of the acid EOS pretreated substrates (*EOS* ethanol organosolv pretreatment, *2N* 2-naphthol, *NS* 2-naphthol-7-sulfonate, *MT* mannitol, *SA* syringic acid)

In lignocellulosic biomass, lignin is linked covalently to hemicellulose, surrounding cellulose microfibrils [24, 25], preventing cellulose accessibility to cellulase enzymes and restricting enzymatic hydrolysis of cellulose. When the raw biomass consisting of 43.81% cellulose, 23.33% hemicellulose and 28.92% lignin (Fig. 1) was directly subjected to enzymatic hydrolysis, cellulose hydrolysis yield was found to be lower than 10% (Fig. 2), indicating a poor hydrolysis on the raw biomass.

Organosolvolysis was suggested to be one of the most efficient methods for boosting biomass saccharification since it could achieve an efficient fractionation of three major constituents of lignocellulose into separated fractions [26]. Thereby, ethanol organosolv (EOS) pretreatment with acid catalyst was performed, yielding a cellulose-enriched residue for subsequent enzymatic hydrolysis (Fig. 1). When acid concentration increased from 10 to 20 mM, lignin removal was enhanced from 22.23 to 59.87%, along with the inevitable solubilization of carbohydrate particularly the hemicellulose components, resulting in an improved delignification selectivity from 0.85 to 1.02 (Table 1). As a result of lignin removal and carbohydrate solubilization, cellulose accessibility evaluated as DR28 adsorption increased from 84.83 of the raw biomass to 175.19 mg g^−1^, contributing to the greater ease of cellulose hydrolysis (Fig. 2).

**Table 1:** Effect of acid addition in ethanol organosolv pretreatment (EOS) on carbohydrate recovery of the pretreated solid, lignin removal and biomass saccharification (data present in average ± stand deviation)

	Cellulose recovery (%)^a^	Hemicellulose recovery (%)^a^	Hemicellulose solubilization (%)	Lignin removal (%)	Delignification selectivity^b^	Cellulose accessibility (mg g^−1^)
Raw biomass	-	-	-	-	-	84.83 ± 4.12
EOS-10 mM	99.33 ± 0.05	68.68 ± 0.25	31.32 ± 0.25	22.23 ± 1.05	0.85 ± 0.04	101.11 ± 3.33
EOS-15 mM	98.83 ± 2.83	59.63 ± 1.03	40.37 ± 1.03	33.07 ± 1.09	0.97 ± 0.07	130.87 ± 3.33
EOS-20 mM	93.57 ± 0.80	38.40 ± 0.59	61.60 ± 0.59	59.87 ± 0.01	1.02 ± 0.03	175.19 ± 2.32
EOS-25 mM	89.40 ± 2.01	34.25 ± 0.75	65.75 ± 0.75	63.77 ± 0.44	0.92 ± 0.04	187.45 ± 5.22

-: not applicable

^a^Recovery was the percentage of original cellulose retained in the pretreated solid

^b^Delignification selectivity was defined as gram lignin removed per gram carbohydrate (cellulose and hemicellulose) solubilized during pretreatment

It was worth noting that, when acid concentration further increased from 20 to 25 mM, lignin content hardly decreased (Fig. 1). Reasons behind this phenomenon might be the repolymerization of lignin and/or the formation of pseudo-lignin. As reported, the formed lignin carbocation intermediates under acidic conditions could attack the adjacent aromatic rings or condensate with another aromatic ring, leading to the formation of repolymerized insoluble lignin, which impaired lignin removal from biomass [27]. Besides, lignin could condense with polysaccharides degradation products [28], leading to the formation of pseudo-lignin and increasing the total mass of residual lignin. As a result, delignification selectivity decreased from 1.02 to 0.92 (Table 1). In addition, the increment of acid concentration from 20 to 25 mM marginally promoted cellulose accessibility (Table 1), making 20 mM to be a reasonable acid concentration for organosolv pretreatment.

### Effect of additives on improving pretreatment efficacy

In order to further improve the efficacy of acid EOS pretreatment, various additives were added, aiming at scavenging the reactive lignin intermediates such as lignin carbocation and/or radical intermediates and mitigating lignin inhibition on subsequent cellulose hydrolysis [1, 29]. The addition of 2-naphthol (2N) slightly increased the lignin content from 18.32 to 20.26% (Fig. 1), while the lignin removal decreased from 59.87 to 55.68% (Table 2). Results agreed with previous work that the 2-naphthol addition increased the total mass of lignin in dilute acid pretreated poplar [30], likely due to the effective incorporation of 2-naphthol to lignin structure, which occupied the reactive carbocation of lignin and prevented further lignin repolymerization reactions [21, 31]. Although no further lignin removal was obtained after 2-naphthol addition, enzymatic hydrolysis yield of cellulose was significantly increased from 44.05% of EOS-20 mM substrate without additive to 71.19% after 2-naphthol addition in pretreatment (20 mM + 2N, Fig. 2). Result suggested higher lignin content did not necessarily predict poorer enzymatic hydrolysis, and physiochemical properties of lignin might play an important role in affecting cellulose hydrolysis.

**Table 2:** Effect of additives on pretreatment efficacy and characteristics of pretreated substrates (*EOS-20 mM* ethanol organosolv pretreatment with 20 mM sulfuric acid, *2N* 2-naphthol, *NS* 2-naphthol-7-sulfonate, *MT* mannitol, *SA* syringic acid)

	Cellulose recovery (%)	Hemicellulose recovery (%)	Lignin removal (%)	Delignification selectivity	Fiber swelling (WRV)	BET surface area (m^2^ g^−1^)	Cellulosic surface area (m^2^ g^−1^)^a^
EOS-20 mM	93.57 ± 0.80	38.40 ± 0.59	59.87 ± 0.01	1.02 ± 0.03	1.82 ± 0.14	3.86	184.83 ± 2.45
20 mM + 2N	94.68 ± 1.79	37.37 ± 0.30	55.68 ± 1.36	0.95 ± 0.11	1.70 ± 0.22	9.01	210.15 ± 4.67
20 mM + NS	93.08 ± 1.26	35.30 ± 0.45	76.13 ± 0.07	1.20 ± 0.01	2.48 ± 0.22	7.80	234.56 ± 7.37
20 mM + MT	94.42 ± 0.39	42.94 ± 0.28	59.49 ± 1.08	1.09 ± 0.02	2.03 ± 0.08	6.99	214.33 ± 2.81
20 mM + SA	93.60 ± 0.21	38.96 ± 0.64	59.53 ± 2.71	1.02 ± 0.10	2.04 ± 0.14	6.94	200.97 ± 2.71

^a^Cellulosic surface area was indicated by the maximum adsorption of DR28 to the pretreated solid, and 1 g of adsorbed dye corresponds to 1055 m^2^ surface

It was interesting to note that, the addition of 2-naphthol-7-sulfonate (NS) obviously facilitated biomass fractionation (Fig. 1), as lignin removal was increased from 59.87 to 76.13%, leading to the highest delignification selectivity of 1.20 (Table 2). The improved lignin removal was probably because the addition of 2-naphthol-7-sulfonate resulted in significantly destructed inter-unit linkages of lignin [32]. As a result, the greatest lignin removal largely helped overcome biomass recalcitrance, leading to the highest cellulose hydrolysis yield of 81.33% for EOS + NS substrate (Fig. 2), which was increased by 85% as compared to that of acid EOS pretreated substrate without additives (44.05% of cellulose hydrolysis yield).

The addition of mannitol (MT) in acid EOS pretreatment improved cellulose hydrolysis yield from 44.05 to 64.19% (Fig. 2), which was increased by 46%. It was likely because mannitol served as carbocation scavenger to form Cα-etherified lignin with plenty hydroxyl groups, like other reported alcohols [16, 18]. Moreover, mannitol could also act as radical scavenger to specially remove **·**OH radicals [23], which would alleviate the radical coupling of lignin fragments during acid organosolv pretreatment [1]. Besides, cellulose hydrolysis yield of acid EOS with syringic acid (SA) treated substrate was improved from 44.05 to 59.36%, which was increased by 35%. Unlike 2-naphthol and 2-naphthol-7-sulfonate that are petroleum-based phenols and currently cannot be obtained from renewable sources [18, 31], mannitol is one of the most abundant compounds in natural macroalgae [33], while syringic acid can be obtained through lignin degradation [22], enabling mannitol and syringic acid as novel green additives in acid EOS pretreatment for a sustainable biorefinery process.

### Effect of additives on mitigating surface barrier effect of lignin

**Fig. 3:**
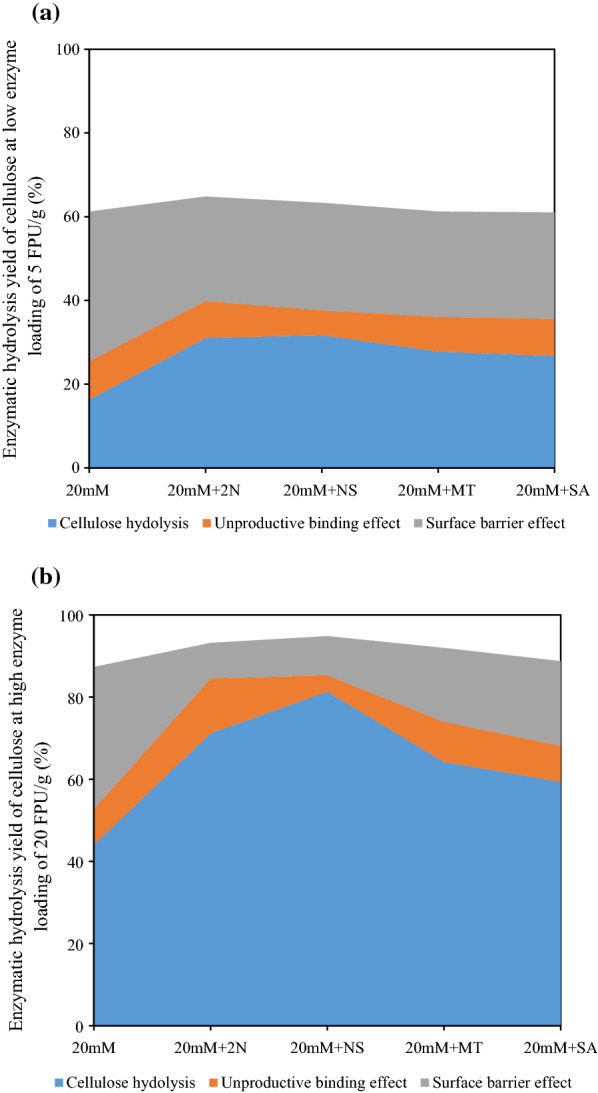
Effect of additives on mitigating lignin inhibition including unproductive binding and surface barrier effect of lignin on cellulose hydrolysis at **a** low enzyme loading and **b** high enzyme loading (*20 mM* ethanol organosolv pretreatment with 20 mM sulfuric acid, *2N* 2-naphthol, *NS* 2-naphthol-7-sulfonate, *MT* mannitol, *SA* syringic acid)

In order to verify the lignin inhibition mitigating effect of additives, extensive delignification and BSA treatment were performed on the EOS substrates, respectively, followed by enzymatic hydrolysis. Delignification treatment by sodium chlorite was believed to eliminate overall lignin inhibition (i.e., unproductive binding and surface barrier effect) on subsequent enzymatic hydrolysis, while the BSA treatment was considered to selectively prevent the unproductive adsorption of lignin [27]. Thus, the difference between hydrolysis of BSA-treated and untreated substrate implied the unproductive binding effect of lignin, while the gap between hydrolysis of delignified and BSA-treated substrate indicated the surface barrier effect of lignin on cellulose hydrolysis [34] (Fig. 3). It was clear that, the unproductive binding effect of lignin was marginally affected by additives, at either low enzyme loading of 5 FPU g^−1^ cellulose (Fig. 3a) or high enzyme loading of 20 FPU g^−1^ (Fig. 3b). However, the surface barrier effect of lignin was more pronouncedly influenced. As illustrated, the surface barrier effect of lignin caused 35.77% reduction in cellulose hydrolysis yield at low enzyme loading of 5 FPU g^−1^, while the reduction was mitigated to 24.94%, 25.68%, 25.14% and 25.40% by the addition of 2N, NS, MT and SA, respectively (Fig. 3a). Furthermore, the surface barrier effect of lignin on cellulose hydrolysis was largely diminished from 34.61 to 8.65%, 9.53%, 17.97% and 20.57%, respectively, at high enzyme loading of 20 FPU g^−1^ (Fig. 3b). The mitigated surface barrier effect of lignin was probably because that the additives in pretreatment changed the physiochemical properties of lignin such as surface chemistry, surface area and surface morphology, favoring the modification of lignocellulosic matrix, thereby influencing cellulose hydrolysis.

#### Surface chemistry (FTIR and XPS analysis)

ATR-FTIR was used to study the surface chemistry of acid EOS pretreated substrates (Additional file 1: Figure S1; Table 3). Relative absorbance for each band was calculated as the ratio of the band intensity of different groups to that of C–H vibration of the aromatic ring at 1510 cm^−1^ [27, 35]. The band at 1329 cm^−1^ indicated that the condensed lignin structure was reduced by additives [36], implying the effectiveness of all used additives in suppressing lignin repolymerization.

**Table 3:** List of chemical bands and the relative absorbance of lignocellulose in ATR-FTIR after acid EOS pretreatment with and without additives (*EOS-20 mM* ethanol organosolv pretreatment with 20 mM sulfuric acid, *2N* 2-naphthol, *NS* 2-naphthol-7-sulfonate, *MT* mannitol, *SA* syringic acid)

Wavenumber	Band assignment	EOS-20 mM	20 mM + 2 N	20 mM + NS	20 mM + MT	20 mM + SA
750 cm^−1^	Naphthalenes	0.07 ± 0.00	0.58 ± 0.04	0.11 ± 0.01	0.10 ± 0.02	0.08 ± 0.01
815 cm^−1^	Naphthalenes	0.20 ± 0.00	0.66 ± 0.03	0.21 ± 0.00	0.23 ± 0.02	0.22 ± 0.00
1329 cm^−1^	S ring plus G ring condensed (i.e., G ring substituted in C_5_)	0.89 ± 0.04	0.73 ± 0.01	0.82 ± 0.00	0.82 ± 0.01	0.83 ± 0.02
1665 cm^−1^	Conjugated carbonyl	0.31 ± 0.00	0.29 ± 0.01	0.41 ± 0.00	0.29 ± 0.02	0.38 ± 0.00
1710 cm^−1^	C=O stretching in unconjugated ketones and carboxyl groups	0.41 ± 0.01	0.34 ± 0.01	0.45 ± 0.03	0.42 ± 0.01	0.43 ± 0.01
2840 cm^−1^	C–H stretching (CH_3_ and CH_2_)	0.37 ± 0.01	0.35 ± 0.01	0.40 ± 0.00	0.44 ± 0.02	0.36 ± 0.01
2940 cm^−1^	C–H stretching (CH_3_ and CH_2_)	0.48 ± 0.01	0.41 ± 0.00	0.51 ± 0.01	0.54 ± 0.01	0.48 ± 0.00
3460 cm^−1^	O–H stretching in aliphatic and phenolic –OH	0.45 ± 0.01	0.41 ± 0.01	0.52 ± 0.00	0.52 ± 0.01	0.46 ± 0.01

After the addition of 2-naphthol in acid EOS pretreatment, a strong strengthening of IR signals at 750 and 815 cm^−1^ was observed (Table 3), which was characteristic for 1,2-disubstituted naphthalenes, proving the incorporation of 2-naphthol [21]. In the case of 2-naphthol-7-sulfonate addition, the intensity of carbonyl groups at 1665, 1710 cm^−1^ and O–H stretching at 3460 cm^−1^ increased (Table 3), likely because 2-naphthol-7-sulfonate additive facilitated lignin depolymerization during acid EOS pretreatment, increasing the carboxylic groups [32]. The addition of mannitol improved the intensity of bands at 2840, 2940 cm^−1^ (CH_2_) and 3460 cm^−1^ (-OH). As reported, alcohols could act as nucleophiles and react with the lignin carbocation intermediates to form Cα-etherified lignin [16], thereby increasing the hydroxyl and methylene groups. In terms of syringic acid addition, notable increment in conjugated carbonyl groups (1665 cm^−1^) were detected (Table 3), primarily due to the incorporation of syringic acid to lignin structure.

High-resolution XPS analysis could also gave information about the elements and types of bonds present at sample surface (Table 4). Subpeaks in C_1s_ at 284.7, 286.6 and 288.4 eV correspond to C_1_ (C–C, C−H or C=C), C_2_ (C–S, C–OH or C–O–C) and C_3_ (O–C–O or C=O), respectively, while subpeaks in O_1s_ at 531.3, 532.4 and 533.3 eV correspond to O_1_ (O–C = O and Ar–O–Ar), O_2_ (C–O–, C=O, C–O–C and O–C=O), O_3_ (Ph–O), respectively.

**Table 4:** Surface lignin coverage and percentage of various bonds or functional groups on fiber surface determined by XPS analysis (*EOS-20 mM* ethanol organosolv pretreatment with 20 mM sulfuric acid, *2N* 2-naphthol, *NS* 2-naphthol-7-sulfonate, *MT* mannitol, *SA* syringic acid)

	Lignin coverage (XPS)	C_1_ (%)^a^	C_2_ (%)^a^	C_3_ (%)^a^	C_1_/C_2_	O_1_ (%)^b^	O_2_ (%)^b^	O_3_ (%)^b^
		284.7 eV	286.6 eV	288.4 eV		531.3 eV	532.4 eV	533.3 eV
EOS-20 mM	0.72	41.78	45.42	12.80	0.92	26.75	32.86	40.40
20 mM + 2 N	0.91	37.15	44.50	18.35	0.83	31.69	45.01	23.30
20 mM + NS	0.54	26.06	51.73	22.21	0.50	36.32	33.14	30.54
20 mM + MT	0.59	28.27	50.70	21.03	0.56	36.82	36.08	27.09
20 mM + SA	0.61	24.99	48.54	26.47	0.51	36.52	35.57	27.91

^a^Subpeaks in C_1s_ correspond to C_1_ (C–C, C−H or C=C), C_2_ (C–S, C–OH or C–O–C) and C_3_ (O–C–O or C=O), respectively

^b^Subpeaks in O_1s_ correspond to O_1_ (O–C=O and Ar–O–Ar), O_2_ (C–O–, C=O, C–O–C and O–C=O), O_3_ (Ph–O), respectively

It was noted that the content of C_1_ subpeak at 284.7 eV was decreased by additives, with a simultaneous increase in C_2_ subpeak at 286.6 eV (Table 4). This was probably because these additives suppressed the lignin repolymerization reactions, reducing the C–C bonds while enhancing the C–O–C linkages [37]. Moreover, XPS results indicated that functional groups were effectively incorporated to lignin, e.g., the sulfonic groups of 2-naphthol-7-sulfonate and hydroxyl groups of mannitol, which largely increased the content of C–S or C–OH in C_2_ subpeak. The carboxylic groups of syringic acid also gave rise to the increase of both C_2_ (C–OH) and C_3_ (C=O) subpeaks. It was suggested the suppression of lignin repolymerization, in combination with the introduction of hydrophilic functional groups, decreased the hydrophobic property of fiber surface, as evidenced by reduced C_1_/C_2_ ratio [38]. In addition, the phenolic oxygen (PhOH), attributed to O_3_ with a binding energy of ~ 533.3 eV [37], was reduced by these additives (Table 4), which could diminish the hydrogen bonding between lignin and cellulase enzymes [17]. Both the decreased surface hydrophobicity and the reduced PhOH content of surface lignin had the potential to alleviate the unproductive binding of cellulase enzymes to surface lignin, thereby diminishing the surface barrier effect of lignin (Fig. 3). Interestingly, a recent work on lignocellulosic fractionation pretreatment using tetrahydrofuran–water co-solvent revealed that the lignin globules deposited on the cellulose surface did not adsorb as much enzyme as lignin in the lignin–carbohydrate complex [9, 39]. In other words, the deposited lignin merely provided an exterior physical obstacle on fiber surface, rather than strong adsorbents to cellulase enzymes [40], which allowed access of enzymes to cellulose with high-efficiency hydrolytic performance [41]. In this work, the notable decreased PhOH groups and hydrophobicity of surface lignin due to additives (Table 4) were believed to be the main reasons for the diminished inhibition of surface lignin.

#### Surface area (dye staining and BET method)

The surface area of pretreated biomass was determined by dye staining and BET method (Table 2), in which the DR28 dye staining method was a measure of the cellulosic surface area, while the BET area was an indicator to reflect the total surface area [42].

It was apparent that the addition of 2-naphthol-7-sulfonate was associated with the highest cellulosic surface area (Table 2), probably due to the highest lignin removal that exposed more fiber surface. And also, the selective lignin removal of 20 mM + NS treatment alleviated lignin’s restrict on fiber swelling as indicated by higher water retention value (WRV; Table 2), contributing to the improved cellulosic surface area.

As mentioned above, the addition of 2-naphthol in acid EOS pretreatment caused a greater lignin content (Fig. 1) and reduced lignin removal (Table 2), which restricted fiber swelling (Table 2). Surprisingly, evidently greater BET surface and cellulosic surface area was obtained in 20 mM + 2 N substrate as compared to that pretreated without additives (EOS-20 mM; Table 2). This phenomenon was probably because, in acidic pretreatments at temperatures above the lignin glass transition temperature, lignin could melt, migrate and redeposit on fiber surface. The additive 2-naphthol had been proved to efficiently suppress lignin repolymerization [20, 21], and the less repolymerized lignin had been reported to be more fluidized and more easily relocated from interior lignocellulosic matrix to fiber surface [43], resulting in greater interior surface area for enzyme attack.

It was also interesting to find that, the mannitol and syringic acid addition led to enlarged surface area as compared to that without additives (EOS-20 mM), despite the lignin content of pretreated substrate and lignin removal were similar (Fig. 1, Table 2). This phenomenon was believed to be closely related to the functional groups incorporation by these additives, such as the hydroxyl and carboxylic groups, as discussed above, which had the potential to improve fiber swelling (Table 2), accounting for the greater surface area than EOS-20 mM substrate. The largely increased surface area contributed to the diminished surface barrier of lignin after the supplementation of these additives in acid EOS pretreatment.

#### Surface morphology (SEM and XPS analysis)

**Fig. 4:**
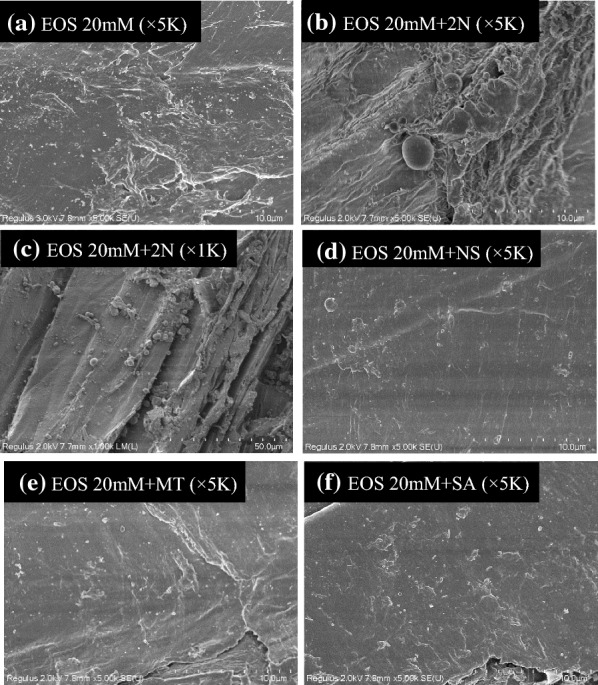
SEM images of acid EOS pretreated substrates without or with additives (*EOS-20 mM* ethanol organosolv pretreatment with 20 mM sulfuric acid, *2N* 2-naphthol, *NS* 2-naphthol-7-sulfonate, *MT* mannitol, *SA* syringic acid)

The surface morphology was investigated by SEM observations (Fig. 4). It was shown that the surface of acid EOS substrate without additives was relatively clean with tiny droplets over the biomass surface (Fig. 4a), since organic solvents were believed to enhance lignin solubility and prevent lignin precipitation [44]. However, the addition of 2-naphthol in EOS pretreatment caused lignin coalescence to form significantly larger lignin droplets (around 1–2 μm, Fig. 4b) and aggravated lignin redeposition on fiber surface (Fig. 4c). In addition, the lignin coverage on fiber surface of EOS + 2N substrate was also quantitatively determined by O/C ratio of XPS analysis [45], which was even higher than that on EOS substrate surface (Table 4). Results confirmed the aggravated exterior lignin coverage caused by 2-naphthol addition in acid EOS treatment, which in turn proved the greater fluidity of the less repolymerized lignin that modified by 2-naphthol. As discussed above, the lignin deposition on fiber surface probably cancelled out the negative effects of residual bulk lignin in the lignin–carbohydrate complex by reducing the unproductive binding, which turned out to be beneficial to enzymatic hydrolysis [9, 40].

The addition of 2-naphthol-7-sulfonate, mannitol and syringic acid led to distinctly cleaner surface when comparing with the surface of EOS-20 mM and 20 mM-2N substrates (Fig. 4). This was mainly because the introduction of hydrophilic functional groups enhanced the solubility of lignin fragments, preventing the lignin redeposition on fiber surface [16]. In particular, the lowest lignin coverage on fiber surface of 20 mM-NS substrate was detected by XPS analysis (Table 4) and observed in SEM image (Fig. 4d). It was likely because sulfonic acid was a stronger acid (*pK*_*a*_ = 3), which was more hydrophilic than carboxylic acid (*pK*_*a*_ = 4.8) and aliphatic hydroxyl groups [46]. Thus, the reduction of surface lignin coverage was believed to play a key role in diminishing lignin inhibition on cellulose hydrolysis, especially the surface barrier effect of lignin [43].

Results above demonstrated that the addition of proper additives, like 2-naphthol-7-sulfonate, during acid organosolv pretreatment could largely facilitate biomass fractionation and improve the ease of biomass saccharification. Detailed analyses on surface chemistry, surface area and surface morphology indicated that proper additives could give rise to the form of less repolymerized surface lignin, which decreased the unproductive binding of cellulase enzymes to surface lignin. In addition, the supplementation of additives (e.g., NS, MT and SA) resulted in a simultaneously increased surface area and decreased lignin coverage. All these factors contributed to the diminished surface barrier effect of lignin, thereby improving the ease of enzymatic hydrolysis of cellulose.

### Lignin adsorbents for textile dye removal from wastewater

In a biorefinery process, the main goals of the pretreatment are to enhance the biomass fractionation and to help recover cellulose, hemicellulose and lignin components in usable forms for downstream utilization [47]. As lignin component contained various functional groups like aliphatic hydroxyl, phenolic hydroxyl and carboxylic groups that could interact with dye molecules, one promising strategy was to use them as adsorbents for dye removal. On the other hand, large-scale use of dyes in textile industry produced a large amount of dye-containing wastewater. Dye wastewater was recalcitrant towards purification and treatment, due to the high organic concentration, toxicity, complex composition, and poor degradability of the dyes [48]. Congo Red (CR) is one of the most commonly used dyes in textile industry [49], which is hazardous to living organisms due to its carcinogenicity and teratogenicity [50]. Among the various physical, chemical, and biological methods, adsorption has been found to be an efficient and economic process to remove dyes. Thus, lignin recovered from acid EOS pretreatment assisted by 2-naphthol-7-sulfonate (20 mM + NS) was evaluated as adsorbents for CR at pH 6, 25 °C and lignin dosage of 10 mg mL^−1^.

As illustrated (Additional file 1: Figure S2), the maximal adsorption (*q*_m_) of CR to ethanol organosolv lignin (EOL) and enzymatic hydrolysis residue (EHR) was determined as 45.87 and 103.09 mg g^−1^, respectively, according to Langmuir model. The adsorption of CR to lignin adsorbents might be due to interactions like hydrogen bonding between the carboxylic and hydroxyl groups of lignin and the sulfonic groups of CR [51], as well as some extent of physical adsorption [52]. The greater adsorption capacity of enzymatic hydrolysis residue (EHR) towards Congo red was likely because the presence of residual cellulose, which possessed electrostatic attraction, hydrogen bonding and hydrophobic interactions with CR [53]. Results indicated that lignin recovered from acidic organosolv pretreatment assisted by proper additive (e.g., 2-naphthol-7-sulfonate) and subsequent enzymatic hydrolysis were favorable adsorbents for textile dye removal from wastewater. In addition to dyes removal, lignin had been utilized as adsorbents for heavy metals removal, gaseous pollutants capture and noble metals recovery [54, 55]. In particular, ethanol organosolv lignin could be applied in production of biopolymers [56] and bioplastics [57] due to its unique properties like rigid chemical structure, thermal stability, antioxidant, antifungal and antibacterial activities.

### Mass balance analysis

**Fig. 5:**
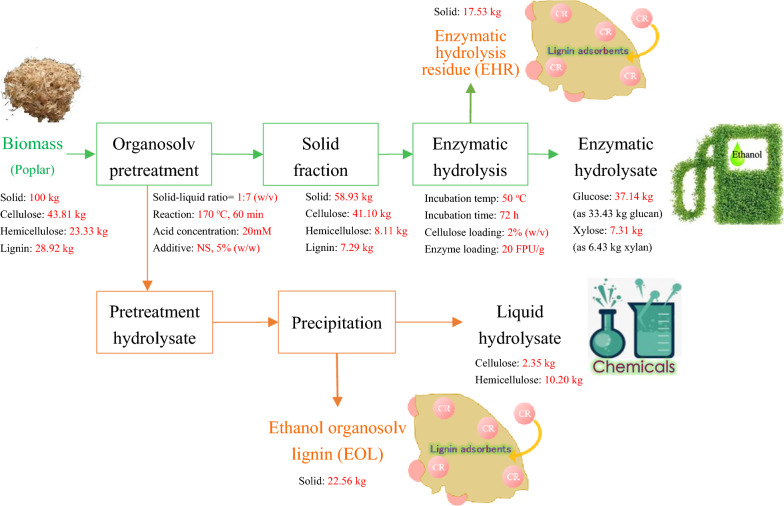
Mass balance of acid EOS pretreatment with the addition of 2-naphthol-7-sulfonate (NS) to coproduce fermentable sugars and lignin adsorbents

To evaluate the overall production of acid EOS pretreatment-based biorefinery process, mass balance analysis was performed (Fig. 5). The acid-catalyzed organosolv pretreatment with 2-naphthol-7-sulfonate addition facilitated biomass fractionation by solubilizing and recovering hemicellulose and lignin components in the liquid fraction, while leaving the accessible cellulose and partial hemicellulose in solid fraction. As illustrated in Fig. 5, 33.43 kg glucan and 6.43 kg xylan could be converted to fermentable sugars after pretreatment on 100 kg dry poplar and subsequent enzymatic hydrolysis, while 2.35 kg cellulose and 10.20 kg hemicellulose were present in liquid hydrolysate as sugars after lignin precipitation. As a result, totally 52.41 kg carbohydrate (i.e., the sum of 35.78 kg cellulose and 16.63 kg hemicellulose) was released and recovered in usable forms after pretreatment and enzymatic hydrolysis, providing a sugar yield of 78%. Moreover, lignin could be extensively solubilized through pretreatment and recovered in the liquid fraction (22.56 kg solid, Fig. 5) with adsorption capacity for Congo red of 45.87 mg g^−1^, which was competitive in comparison with other biomass-based adsorbents or modified lignin in previous works [50]. The enzymatic hydrolysis residue (17.53 kg), mainly consisting of cellulose and lignin, showed even better adsorption to Congo red, with a value of 103.09 mg g^−1^. The utilization of lignin as adsorbents for textile dye removal from wastewater could not only diversify the final products, but also enable the holistic utilization of wastes to expand the profit margin as a part of economical and sustainable biorefinery processes.

## Conclusions

The biorefinery process based on acidic organosolv pretreatment was proved to enable coproduction of fermentable sugars and lignin adsorbents, allowing the holistic utilization of lignocellulosic biomass for sustainable biorefinery. During this process, the proper additives, in particular mannitol and syringic acid that served as novel green additives, could efficiently improve enzymatic digestibility of biomass. Up to 78% of the carbohydrates could be released and recovered after pretreatment and enzymatic hydrolysis. Both the ethanol organosolv lignin and enzymatic hydrolysis residue exhibited as promising adsorbents for textile dye removal from wastewater.

## Materials and methods

### Materials

Poplar sawdust, with a moisture content of 6.76 ± 0.18%, was acquired from Xuzhou, Jiangsu Province, China. Chemicals including 2-naphthol (2N), 2-naphthol-7-sulfonate (NS), mannitol (MT), syringic acid (SA) and ethanol were analytical reagents (AR, > 99% purity). These reagents and sulfuric acid (H_2_SO_4_, 95–98 wt%) were obtained from Sinopharm Chemical Reagent Co., Ltd.

### Ethanol organosolv pretreatments

Ethanol organosolv (EOS) pretreatment was performed as follows: 100 g dry mass of poplar was soaked in an aqueous solution (ethanol–water ratio of 65:35) containing 10, 15, 20 and 25 mM H_2_SO_4_ at a solid-to-liquid ratio of 1:7 (g:mL). The mixture was pretreated at 170 °C for 60 min. To examine the effect of various additives, the biomass was mixed with each additive separately (5% w/w, based on dry biomass) and pretreated at the same conditions. After pretreatment, the reactor was cooled down immediately in cold tap water. The solid fraction was separated from pretreatment hydrolysate by filtration. Deionized water of 2100 mL was used to wash the separated solid. The pretreatment hydrolysate and washing water were collected for precipitation and recovery of lignin, while the washed solid was kept at 4 °C for chemical composition analysis and further use.

### Enzymatic hydrolysis of the EOS substrates

Enzymatic hydrolysis of the EOS substrates was performed in acetate buffer (50 mM, pH 4.8) at 50 °C with shaking at 180 rpm. The enzymes used for the enzymatic hydrolysis of cellulose were Cellic CTec 2 (SAE0020, filter paper activity of 185 FPU g^−1^, 233 mg protein g^−1^) from Sigma-Aldrich China Inc. Enzyme dosage was 5 or 20 FPU of cellulase per gram cellulose, respectively. Enzymatic hydrolysis was conducted at cellulose loading of 2% (w/v) in a 100 mL Erlenmeyer flask. Samples were taken after 72 h hydrolysis. Enzymes were inactivated by heating at 100 °C for 5 min, followed by sugar analysis. All experiments were performed in duplicate.

### Delignification and BSA treatment of the EOS substrates

Prior to enzymatic hydrolysis, the EOS substrates were extensively delignified: 3 g washed solid was mixed with 30 mL solution containing 5% (w/v) sodium chlorite and 1% (v/v) acetic acid. The mixture was incubated overnight at room temperature in the dark for 3 rounds of delignification. Enzymatic hydrolysis of the delignified substrate was performed as specified above. The gap between enzymatic hydrolysis of delignified and non-delignified EOS substrates indicated the total lignin inhibition on cellulose hydrolysis, including unproductive binding effect and surface barrier effect [45].

Another set of treatment was carried out to isolate unproductive binding effect of lignin: prior to the addition of cellulase, bovine serum albumin solution (BSA, 0.1 g mL^−1^, 1 mL) was added to the enzymatic hydrolysis system of the EOS substrate, equal to a final BSA concentration of 5 g L^−1^. The mixtures without cellulase were incubated at 50 °C, 180 rpm for 24 h. After that, cellulase (20 FPU per gram cellulose) was added in each flask, enzymatic hydrolysis was performed. The discrepancy between enzymatic hydrolysis with and without the BSA treatment revealed the unproductive binding effect of lignin [45].

### Preparation of lignin adsorbents

The pretreatment hydrolysate from EOS pretreatment and washing water were collected, as described above. The suspension was stabilized on bench overnight. The precipitated solids were separated from liquid fraction by filtration on Whatman No. 1 filter paper and washed three times with warm water to remove residual acid. In addition, after organosolv pretreatment and subsequent enzymatic hydrolysis, solid residues were collected, which mainly consisted of lignin and leftover cellulose. The solid from pretreatment and enzymatic hydrolysis was oven dried at 105 °C overnight and used as adsorbents for Congo red dye.

### Analytical methods

Chemical components of biomass samples were analyzed by following the method developed by the US National Renewable Energy Laboratory [58]. Briefly, the extractive-free biomass was incubated with 72% (w/w) H_2_SO_4_ at 30 °C for 1 h, and followed by 4% (w/w) H_2_SO_4_ treatment at 121 °C for 1 h. The monomers (glucose, mannose, xylose and arabinose) concentration in the acid hydrolysate was determined by high-performance liquid chromatography (HPLC) system [59]. Cellulose content was calculated based on the glucose concentration, while hemicellulose content was calculated based on the sum of mannose, xylose and arabinose. To assess cellulose accessibility, staining method by DR28 was carried out as described elsewhere [60]. All the measurements were performed in duplicate. The elements and types of bonds present at sample surface were evaluated by high-resolution X-ray photoelectron spectroscopy (XPS) system (Thermo Fisher Scientific, Waltham, US). The chemical structure of the samples surface was determined by attenuated total reflection Fourier transform infrared spectra (ATR-FTIR, Spectrum Two, PerkinElmer, US). Each sample was analyzed in its dry form. Spectra of each sample ranging from 650 and 4000 cm^−1^ were averaged from 64 scans at a spectral resolution of 4 cm^−1^. The surface morphology changes of poplar samples were observed by scanning electron microscope (SEM) at magnifications of 5K and 1K. Porosity, including specific surface area, pore size and pore volume, was evaluated by Brunauer–Emmett–Teller (BET) method using nitrogen adsorption/desorption at 77K with Nova Station (Quantachrome Instruments).

## Supplementary Information

**Additional file 1: Figure S1.** ATR-FTIR spectra of acid EOS pretreated substrate without or with additives (EOS-20mM: ethanol organosolv pretreatment with 20 mM sulfuric acid; 2N: 2-naphthol; NS: 2-naphthol-7-sulfonate; MT: mannitol; SA: syringic acid). **Figure S2.** Adsorption of Congo red (CR) to lignin adsorbents (obtained from acid EOS pretreatment with the addition of 2-naphthol-7-sulfonate) at 25 °C, pH 6.0, lignin adsorbents dosage of 10 mg/mL (EHR: enzymatic hydrolysis residue; EOL: ethanol organosolv lignin).

## Data Availability

All data generated or analyzed during this study are included in this published article and its Additional file 1.

## Abbreviations

EOS: Ethanol organosolv pretreatment
2N: 2-Naphthol
NS: 2-Naphthol-7-sulfonate
MT: Mannitol
SA: Syringic acid
BSA: Bovine serum albumin
XPS: X-ray photoelectron spectroscopy
BET: Brunauer–Emmett–Teller
ATR-FTIR: Attenuated total reflection Fourier transform infrared spectra
HPLC: High-performance liquid chromatography
