# Supplementary material for: Organosolv pretreatment assisted by carbocation scavenger to mitigate surface barrier effect of lignin for improving biomass saccharification and utilization
Source: Biotechnol Biofuels. 2021 Jun 12;14:136. doi: 10.1186/s13068-021-01988-w (PMC8199801; doi:10.1186/s13068-021-01988-w)
Supplement: Supplementary file 1 — Additional file 1: Figure S1. ATR-FTIR spectra of acid EOS pretreated substrate without or with additives (EOS-20mM: ethanol organosolv pretreatment with 20 mM sulfuric acid; 2N: 2-naphthol; NS: 2-naphthol-7-sulfonate; MT: mannitol; SA: syringic acid). Figure S2. Adsorption of Congo red (CR) to lignin adsorbents (obtained from acid EOS pretreatment with the addition of 2-naphthol-7-sulfonate) at 25 °C, pH 6.0, lignin adsorbents dosage of 10 mg/mL (EHR: enzymatic hydrolysis residue; EOL: ethanol organosolv lignin). [file 13068_2021_1988_MOESM1_ESM.docx]

**Figures caption:**

**Fig. S1** ATR-FTIR spectra of acid EOS pretreated substrate without or with additives (EOS-20mM: ethanol organosolv pretreatment with 20 mM sulfuric acid; 2N: 2-naphthol; NS: 2-naphthol-7-sulphonate; MT: mannitol; SA: syringic acid).

**Fig. S2** Adsorption of congo red (CR) to lignin adsorbents (obtained from acid EOS pretreatment with the addition of 2-naphthol-7-sulphonate) at 25 ^o^C, pH 6.0, lignin adsorbents dosage of 10 mg/mL (EHR: enzymatic hydrolysis residue; EOL: ethanol organosolv lignin).

**Fig. S1**

**Fig. S2**
